# Effectiveness of imaging genetics analysis to explain degree of depression in Parkinson’s disease

**DOI:** 10.1371/journal.pone.0211699

**Published:** 2019-02-11

**Authors:** Ji Hye Won, Mansu Kim, Bo-yong Park, Jinyoung Youn, Hyunjin Park

**Affiliations:** 1 Department of Electrical and Computer Engineering, Sungkyunkwan University, Suwon, Korea; 2 Center for Neuroscience Imaging Research, Institute for Basic Science, Suwon, Korea; 3 Department of Neurology, Sungkyunkwan University School of Medicine, Samsung Medical Center, Seoul, Korea; 4 Neuroscience Center, Samsung Medical Center, Seoul, Korea; 5 School of Electronic and Electrical Engineering, Sungkyunkwan University, Suwon, Korea; Shenzhen University, CHINA

## Abstract

Depression is one of the most common and important neuropsychiatric symptoms in Parkinson’s disease and often becomes worse as Parkinson’s disease progresses. However, the underlying mechanisms of depression in Parkinson’s disease are not clear. The aim of our study was to find genetic features related to depression in Parkinson’s disease using an imaging genetics approach and to construct an analytical model for predicting the degree of depression in Parkinson’s disease. The neuroimaging and genotyping data were obtained from an openly accessible database. We computed imaging features through connectivity analysis derived from tractography of diffusion tensor imaging. The imaging features were used as intermediate phenotypes to identify genetic variants according to the imaging genetics approach. We then constructed a linear regression model using the genetic features from imaging genetics approach to describe clinical scores indicating the degree of depression. As a comparison, we constructed other models using imaging features and genetic features based on references to demonstrate the effectiveness of our imaging genetics model. The models were trained and tested in a five-fold cross-validation. The imaging genetics approach identified several brain regions and genes known to be involved in depression, with the potential to be used as meaningful biomarkers. Our proposed model using imaging genetic features predicted and explained the degree of depression in Parkinson’s disease appropriately (adjusted R^2^ larger than 0.6 over five training folds) and with a lower error and higher correlation than with other models over five test folds.

## Introduction

Parkinson’s disease (PD) is the second most common neurodegenerative disorder [1]. PD is characterized primarily as a movement disorder, but recent research indicates that a variety of non-motor symptoms including constipation, sleep disturbances, diabetes, cognitive decline, and depression may play a role in PD development [2]. Among these symptoms, depression is the most common non-motor symptom of PD, occurring in around 40–50% of all patients diagnosed with PD [3,4]. Depression can predate symptoms of PD for several years before the worsening of motor symptoms and belongs to the group of non-motor features that might predict the development of PD [5,6]. Depression in PD (DPD) can aggravate all other symptoms, including the worsening of motor symptoms, rapid disease progression, and reduced cognitive function [7]. DPD is one of the major causes of poor quality of life and disability in PD patients [8]. However, DPD has not yet been fully explored [9,10].

The criteria involved in general depression and DPD are subject to bias as they are either psychiatric scales or clinical interviews. Thus, neuroimaging techniques have been used to reduce subjective bias and better understand DPD or general depression. Many studies that have used single photon emission tomography (SPECT) and positron emission tomography (PET) were mainly focused on the dopaminergic and serotonergic systems [11,12]. Magnetic resonance imaging (MRI) techniques have been invaluable for neuroscientists, providing insight into the structure, biochemistry, and function of the living human brain. Diffusion tensor imaging (DTI), a variant of MRI, can quantify the integrity of white matter fiber tracts noninvasively. Recent work using DTI has shown that altered structural white matter connectivity in the fronto-limbic systems distinguishes major depressive disorder (MDD) from healthy controls [13,14]. In this study, we used probabilistic tractography to characterize the regions of interest (ROIs) that affect depression. The fiber information from DTI might be helpful in distinguishing the degree of depression.

However, several factors impede our ability to diagnose a person with a psychiatric condition including depression based on neuroimaging: 1) there is considerable variation in brain imaging among people with the same diagnosis, 2) psychiatric conditions can present quite differently in different individuals, 3) different psychiatric conditions often share similar symptoms, and, finally, 4) similar groups of brain areas are involved in diverse psychiatric conditions. Psychiatric disorders such as depression are difficult to characterize using neuroimaging alone. We tried to overcome this limitation by adopting an imaging genetics approach that uses both neuroimaging and gene data.

Both genetic and environmental factors contribute to individual differences in brain function and behavior [15]. Although it remains unclear to what degree each of these factors contributes to variation in brain function, it is likely that genetics contribute to a significant portion of the variance. Genes that are weakly related to psychiatric disorders such as depression are relatively strongly related to the function of neural systems involved in processing cognitive and emotional information in the brain. There are probably no individual genes for psychiatric disorders, but rather genetic variations that impact the relevant information processing in the brain [16]. The imaging genetics approach is more sensitive than the conventional genome-wide association approach (GWAS) as it integrates imaging information as an intermediate phenotype [16,17]. Ideally, the relationship between the regional pattern of neuroimaging and genetic variants could explain the underlying biological mechanisms of depression or DPD and provide an intuitive concept of DPD mediated by brain systems affected by genetic variants [15].

The aim of this study was to identify genetic features affecting DPD using the imaging genetics approach and to construct an analytical model for predicting the degree of depression in PD. We hypothesized that a neuroimaging genetics approach would be sensitive enough to identify genetic features as biomarkers that could explain the degree of DPD. Furthermore, we constructed other models using imaging features only and genetic features based on references and compared them against our image genetics model.

## Methods

### Subjects and imaging data

This study was a retrospective analysis of anonymized data and institutional review board (IRB) approval was obtained at Sungkyunwkan University. All data were obtained with informed written consent in accordance with established human subject research procedures expressed in the Declaration of Helsinki. Our study was performed in full accordance with the local IRB guidelines. We used diffusion MRI, T1-weighted MRI and DNA genotyping data of 81 patients with PD obtained from the Parkinson’s Progression Markers Initiative (PPMI) database [17]. Patient information, including age, sex, and clinical assessment (e.g. the geriatric depression scale [GDS], Movement Disorder Society sponsored unified Parkinson's disease rating scale [MDS-UPDRS][18]), were collected for each subject at a baseline visit, as shown in Table 1. Additional clinical scores of State Trait Anxiety Inventory (STAI), Montreal Cognitive Assessment (MoCA), and Questionnaire for Impulsive-Compulsive Disorder (QUIP) were collected [19–21]. PD was diagnosed using the criteria established by the PPMI consortium [17]. GDS is a clinical score indicating the degree of depression. Subjects who have a GDS of 6 or more were considered to be depressed patients [22]. We randomly sub-sampled the data so that sex ratio (female to male) was two, which is the sex ratio in PD [23]. Table 1 shows the clinical information of the DPD and non-depressed patients with PD (nDPD) patients in this study.

**Table 1:** Patient information.

	DPD	nDPD	p-value
**Number of subjects**	36	45	-
**Age**	61.63±10.60	63.08±9.83	0.52
**Sex (M:F)**	24:12	30:15	-
**MDS-UPDRS**^a^	34.72±16.33	33.82± 14.04	0.79
**STAI**^b^	67.34±18.17	62.96±16.87	0.27
**MoCA**^c^	27.06±1.80	27.16±2.54	0.84
**QUIP**^d^	0.31±0.58	0.18±0.53	0.28
**GDS**^e^	6.86±1.12	4.58±0.75	<10^−16^

^a^MDS-UPDRS: Movement Disorder Society-sponsored unified Parkinson's disease rating scale.

^b^STAI: State Trait Anxiety Inventory [19].

^c^MoCA: Montreal Cognitive Assessment.

^d^QUIP: Questionnaire for Impulsive-Compulsive Disorder [21].

^e^GDS: geriatric depression scale.

**Fig 1:**
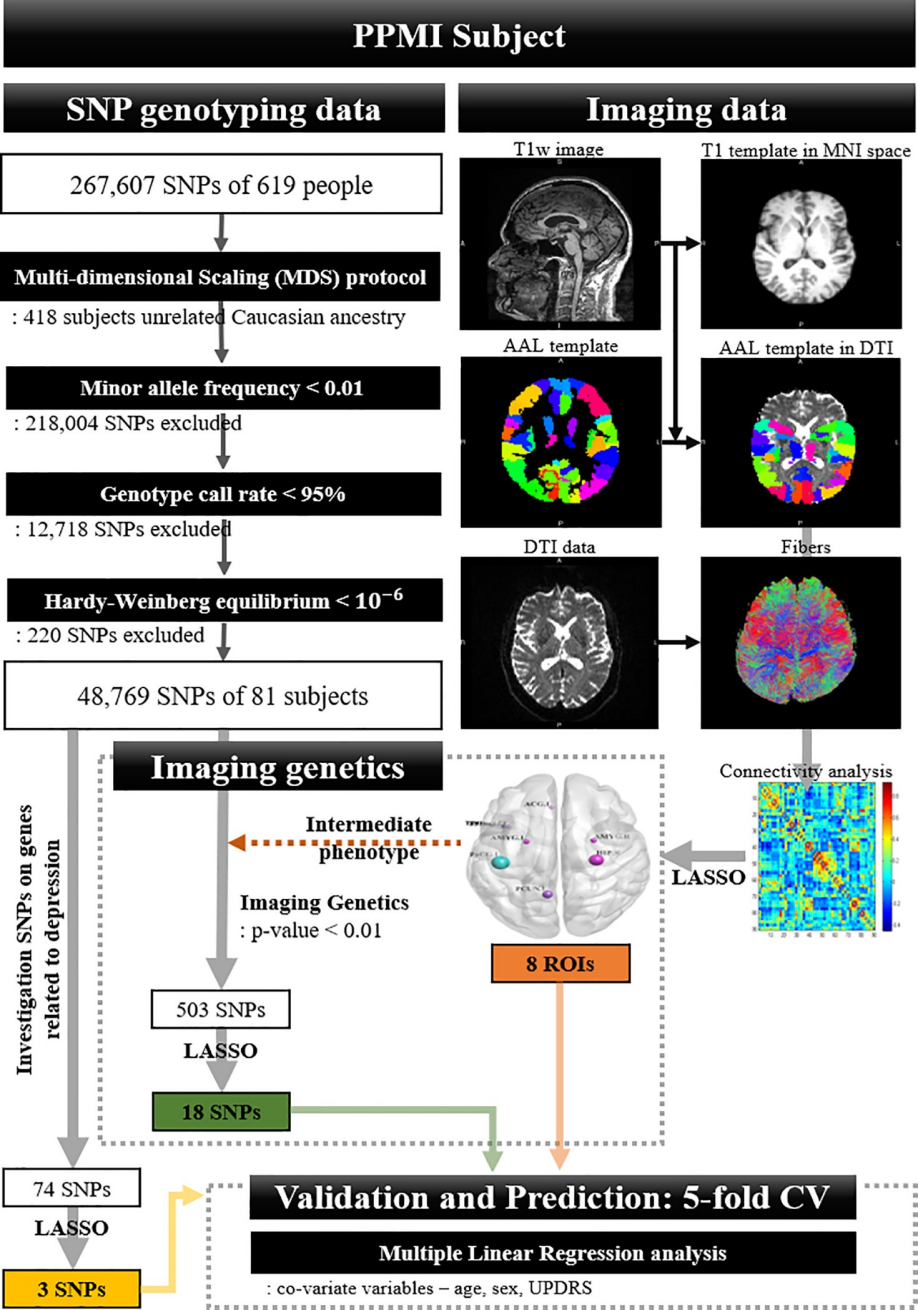
Overview of neuroimaging, reference-based genetics, and imaging genetics processing steps.

Diffusion MRI and T1-weighted MRI data were obtained from the PPMI database [17]. T1-weighted MRI were obtained using the following parameters on a 3T scanner (repetition time [TR] = 2,300 ms, echo time [TE] = 2.98 ms, image matrix = 240 × 256 × 176, and voxel resolution = 1 × 1 × 1 mm^3^). Diffusion MRI were obtained using the following parameters on a 3T scanner (b = 1,000 s/mm^2^, 64 diffusion gradient directions with one b0 image, image matrix = 116 × 116 × 72, and voxel resolution = 1.98 × 1.98 × 2 mm^3^). Our study considered two types of information (i.e., neuroimaging and genetic information) and many procedures are necessary to process them. The schematic of the overall processing steps is given in Fig 1 [24]. Details regarding the procedures are provided later in this study.

### Genetic data and quality control

We obtained DNA samples genotyped by NeuroX genotyping arrays from the PPMI. The following procedure pertains to the genetic data of the PPMI. All available DNA samples were genotyped by using NeuroX as genotyping arrays. Briefly, NeuroX was proposed to cover more than 240,000 exonic variants so that neurodegenerative disease could be studied effectively [25,26]. These neurodegenerative variants influencing specific diseases contain loci. Loci were derived from the maximum completed meta-analyses of PD patients and normal controls (NC), which identified known PD mutations and unusual or high-risk variants [26]. Genotyping was performed according to the Illumina protocol using an Immunochip array [25,26]. The Immunochip is an Illumina Infinium iSelect HD Custom Genotyping array including 195,806 single nucleotide polymorphisms (SNPs) in 196,524 polymorphisms [25–27].

We performed quality control of the genetic data by applying the Enhancing Neuro Imaging Genetics through Meta-Analysis (ENIGMA) protocol [28] using Plink v1.09 software [29]. The ENIGMA protocol included the following processes: 1) call rate check per subject, 2) sex check, 3) sibling pair identification and 4) population stratification using multi-dimensional scaling. In addition, the SNPs that did not meet the quality control criteria (minor allele frequency < 0.01; genotype call rate < 95%; Hardy-Weinberg equilibrium < 10^−6^) were filtered out of the dataset [24,28,29]. SNPs were only kept if they did not belong to the Caucasian population according to the HapMap3 reference population. The age, sex ratio, and MDS-UPDRS of the DPD and nDPD groups were matched (Table 1). The above section is described as the “SNP genotyping data” section of PPMI subject in Fig 1.

### Neuroimaging processing and selection of imaging features

All imaging data were pre-processed using the Functional Magnetic Resonance Imaging of the Brain (FMRIB) Analysis Group Software Library (FSL) [30]. Structural T1 images were skull-stripped and nonlinearly registered onto the common Montreal Neurological Institute (MNI) space. DTI data were corrected for distortion and movement artifacts and subsequently used to perform probabilistic tractography. Head motion and image distortions induced by eddy currents were corrected by applying a 3D full-affine alignment of each image to the mean no-diffusion-weighting (b0) image. DTI data were averaged and concatenated after the correction of distortion. DTI images were co-registered onto the common MNI space as with T1 images, then we adopted the automated anatomical labeling (AAL) atlas defined on the MNI space to specify the ROIs.

Probabilistic tractography algorithm implemented in FSL software was applied to extract the fiber connection in all ROIs [31]. We performed Bayesian Estimation of Diffusion Parameters Obtained using Sampling Techniques (bedpost tool in FSL) [32] on diffusion data, which allows the modeling of crossing fibers within each voxel. Next, we used the probtrackX tool to generate a connectivity distribution between each ROI that guides multiple fiber samples starting from a seed voxel to a specified target region. The algorithm propagated a line from the center of the seed voxel to the direction of not only the dominant ROI but also that of non-dominant ones until the line strayed out. Fiber tracking ended when fiber direction changed rapidly and probabilistic tractography was performed in the native space for each participant. Here, all 90 ROIs were used as seeds. Each brain region was selected as the seed region, and its connectivity probabilities to each of the other 89 regions were calculated. The computed fiber streamlines remained within the masked regions. One thousand fiber streamlines were generated from each voxel within the seed region, and only those that reached the target region were retained as the final white matter connection. The streamlines were terminated once they reached a target region.

Connectivity analysis is a representative method of analyzing complex systems such as the brain, and it uses nodes and edges to analyze a given system. Connectivity analysis requires nodes to be specified so that correlations among nodes can be computed. We considered 90 ROIs specified by the atlas via image co-registration as nodes in a graph [33,34]. Each edge was defined as fiber probability connecting a pair of regions. We applied a weighted and undirected network model to perform connectivity analysis. The constructed graph is commonly referred to as the structural connectivity matrix as it reflects structural connectivity via white matter fiber tracts. We computed the degree centrality (DC), the number of direct connections to all other nodes with respect to a given node, among several network parameters to quantify the structural connectivity [34]. The data used were collected from the 12 centers. We adopted a dummy coding regression model to remove multi-site effects for the DC value [35].

There were many (i.e., 90) DC values computed from the ROIs and thus we adopted the least absolute shrinkage and selection operator (LASSO) algorithm to select regional imaging features that could characterize DPD. The LASSO is a penalized regression model that selects a sparse set of features that can explain a dependent variable. We sought to find imaging features that were related to DPD; thus, the dependent variable was the GDS score. The LASSO requires a smoothness term (*λ*), which was set by minimizing the mean squared residual in a 10-fold cross-validation. Finally, k non-zero beta values were selected as significant imaging features to appropriately explain the GDS. The above section is described as the “Imaging data” section of PPMI subject in Fig 1.

### SNP selection from imaging genetics

We chose the imaging features selected by LASSO variable selection as intermediate phenotypes and performed imaging genetics analysis to detect genetic variants associated with the intermediate phenotype. Plink software was employed for imaging genetics analysis [29]. Linear regression by conventional genetics analysis was used to evaluate the association of allele genotypes with the intermediate phenotypes with covariates included for age and sex. SNPs were identified above a threshold of corrected empirical-p < 0.01 with Bonferroni correction. To provide a sufficient condition for evaluating the association, we considered genetic variants as significant SNPs only if they were identified more than once in different intermediate phenotypes (i.e., more than one ROI from the previous section). This was to improve the consistency of the association between the significant SNPs and the identified imaging features. It is likely that the number of selected SNPs is still excessive (possibly in the hundreds) as with many genetic association studies [36]. We adopted another layer of SNP selection to reduce the number of selected SNPs. Similar to selecting imaging features, we applied the LASSO framework using GDS scores as the dependent variable to select SNPs. The smoothness term (λ) of LASSO was set using the approach described before. The above section is described as the “Imaging genetics” and “SNP genotyping data” sections of PPMI subject in Fig 1.

### Construction of imaging genetics based linear regression model

A five-fold cross-validation was adopted separating the training and the test data. The data were divided into five folds. Four folds were used to train and construct the models and the remaining fold was used to test the constructed models. For each training fold, we constructed a linear model using multiple linear regression based on the features selected from the previous steps to explain the GDS score. Our proposed model was constructed using genetic features from imaging genetics approach that used both the imaging and genetic features. Our regression model used age, sex, and MDS-UPDRS as covariate variables:

Y=α×SNP+β×COV+ε,

where Y was the GDS, SNP were imaging genetics features, COV were co-variants, α and β were estimated coefficient, and ε was the error. The quality of multiple linear regression was assessed with adjusted R^2^ values. We applied our trained model to the left out test fold five times each time using a different test fold. This resulted in five sets of performance metrics of the models, which were averaged to yield a single scalar value. We assessed the performance of the prediction using Pearson’s correlation between actual and predicted GDS. Root mean squared (RMS) error was also used to quantify how well the prediction of the actual GDS worked.

### Construction of other multiple regression models for comparison

We constructed two other linear models to compare with our imaging genetic based model. This process also performed in a five-fold cross-validation fashion as described above.

#### A) Multiple regression model with only neuroimaging features

We constructed a linear model with only neuroimaging features. The imaging features were obtained using the procedure described in “Neuroimaging processing and selection of imaging features” section to compute intermediate phenotypes. These imaging features not new ones for this model but intermediate phenotypes used as part of the imaging genetic analysis.

#### B) Multiple regression model with conventional genetic features

We constructed a linear model with SNPs related to DPD based on references. This approach did not consider imaging genetics approach. Among gene-specific research related to DPD, there were no references. Thus, we investigated genes related to depression. There are 5,992 human genes for depression ranked by a relevance score according to the GeneCards database [37,38]. We chose the top 50 genes from the gene list associated with depression and identified SNPs that were also found in the PPMI data. Similar to the imaging genetic approach, we selected a few SNPs using the LASSO framework. We composed the model in the same way as before using several genetic features resulting from LASSO.

These two other models were compared to our model to show that the features obtained by imaging genetics could better than those obtained with conventional neuroimaging and genetics analysis procedures.

## Results

### Selected imaging features from structural connectivity

We used diffusion MRI, T1-weighted MRI and DNA genotyping data of 81 patients with PD obtained from the PPMI database [17]. Structural connectivity analysis based on probabilistic tractography was performed and we identified imaging features that were significantly related to the degree of depression using the LASSO [39,40]. The selected imaging features were strongly correlated with the GDS score and this does not mean that imaging features between DPD and nDPD would be significantly different. We have reported the DC values of the identified imaging features obtained from the LASSO selection procedure for patients with DPD and nDPD to further demonstrate the potential effectiveness of the selected imaging features (Table 2). The DC values of these eight regions were used as intermediate phenotypes in imaging genetics analysis and were also used to construct the neuroimaging only model.

**Table 2:** The selected imaging features from structural connectivity.

Regions from atlas		Degree centrality		p-value
#	Name	DPD	nDPD	
31	Anterior cingulate and paracingulate gyri (Left)	8.31±8.85	2.43±2.00	0.277
38	Hippocampus (Right)	8.04±9.52	2.78±3.29	0.035
41	Amygdala (Left)	1.66±2.50	0.77±0.90	<10^−4^
42	Amygdala (Right)	2.08±2.32	1.07±1.12	0.343
57	Postcentral gyrus (Left)	6.51±7.22	1.06±1.68	0.022
67	Precuneus (Left)	7.76±8.44	2.07±1.50	0.104
83	Temporal pole: superior temporal gyrus (Left)	2.69±3.49	1.14±0.95	0.001
87	Temporal pole: middle temporal gyrus (Left)	0.86±1.22	0.53±0.55	0.003

DC values are reported as the mean ± standard deviation (SD) format. The values were reported for two groups (DPD and nDPD) to show how the imaging features affect depression in PD.

### Genotype data

We obtained DNA samples genotyped by NeuroX genotyping arrays from the PPMI that were processed using the PPMI protocol. Quality control was performed on the genetic data by applying the ENIGMA protocol [28]. At first, the genotype data contained 267,607 SNPs of 619 people (409 males and 210 females). To reduce the population stratification effect, we used 418 Caucasians from 619 subjects with complete imaging measurements at baseline. We excluded SNPs if they met the criteria: 218,004 SNPs had a minor allele frequency < 0.01, 12,718 SNPs had a genotype call rate < 95%, and 220 SNPs had a Hardy-Weinberg equilibrium < 10^−6^. After frequency and genotype pruning, 48,769 SNPs remained. We then proceeded with imaging genetics approach using only these quality-controlled SNPs.

### Selected SNPs from imaging genetics

We applied the imaging genetics approach using the identified imaging features (Table 2) as intermediate phenotypes to find SNPs. After a Bonferroni-corrected significant threshold of empirical-p < 0.01, more than 500 SNPs per ROI remained. Only 503 SNPs were retained after the SNP filtering which considered SNPs associated with at least two imaging features. After using LASSO selection, we were left with 18 SNPs that described the GDS appropriately (Table 3).

**Table 3:** Selected SNPs from imaging genetics.

SNP	Gene	CHR^a^	Base-pair location	Minor allele	Intermediate phenotype (#)^b^	Asymptotic p-value
exm-rs11265263	*-*	-	-	A	41	0.0005
					42	0.0077
					87	0.0033
exm2261278	*NRXN1*	2	50565462	C	41	0.0004
					42	0.0078
					87	0.0033
exm-rs2629046	*-*	-	-	C	41	0.0017
					83	0.0055
exm2265730	*ARHGAP24*	4	86808963	G	38	0.0011
					42	0.0067
					83	0.0087
exm2266008	*-*	-	-	G	38	0.0027
					42	0.0020
exm-rs6556756	*LOC101927835*	5	163889280	G	57	0.0062
					67	0.0063
NeuroX-rs56107012	*-*	-	-	G	38	0.0043
					42	0.0036
exm847519	*LOXL4*	10	100017453	G	41	0.0002
					87	0.0033
exm883966	*OR52N2*	11	5842310	T	31	0.0003
					87	0.0031
exm952147	*PDGFD*	11	103818395	C	31	0.0074
					83	0.0065
exm1092110	*PCK2*	14	24572932	A	41	0.0046
					83	0.0023
					87	0.0096
exm2272098	*RAB15*	14	65428165	C	57	0.0041
					67	0.0061
exm1179562	*PEAK1*	15	77471361	A	31	0.0212
					38	0.0020
					42	0.0057
exm1185136	*SLC28A1*	15	85478729	A	41	0.0079
					42	0.0007
exm-rs4517902	*LOC284395*	19	29851078	C	42	0.0053
					83	0.0009
					87	0.0029
exm1513594	*ZNF772*	19	57985460	T	38	0.0006
					42	0.0043

SNPs without matching gene-related information, such as chromosome (CHR) and base-pair location, have blank entries. We described only intermediate phenotypes with asymptotic p-values less than 0.01.

^a^ CHR: chromosome.

^b^ Intermediate phenotype (#) refers to the numerical labels of the ROI names in “Regions from atlas” of Table 2. This was done to improve readability of the table.

### Selected SNPs based on references for comparison

We searched for SNPs related to DPD not using the imaging information but using references for comparison. We searched for genes related to depression because there were no references among gene-specific research related to DPD. We found 5,992 genes associated with depression from the GeneCards database, which integrates information about genes, proteins, and disease [37,38]. We chose the top 50 genes sorted by relevance score for depression and 954 SNPs were identified that were also in the PPMI database. We selected three SNPs associated with GDS using the LASSO framework (Table 4). The three SNPs associated with DPD were exm2267347, exm1187499, and exm-rs9303521.

**Table 4:** Selected SNPs from references.

SNP	Gene	CHR ^a^	Base-pair location	Minor allele	GIFtS^b^	Relevance score
exm2267347	*COL2A1*	12	48375568	G	54	18.34
exm1187499	*POLG*	15	89859994	A	52	20.75
exm-rs9303521	*CRHR1*	17	43805194	T	53	17.84

The GeneCards Inferred Functional Score (GIFtS) uses the Genecards annotations to produce scores aimed at predicting the degree of a gene’s functionality. The relevance score is the Novoseek score of the relevance of the disease to the gene based on literature text-mining algorithms.

^a^ CHR: chromosome.

^b^ GIFtS: GeneCards Inferred Functional Score

### Validation and prediction of linear models

**Fig 2:**
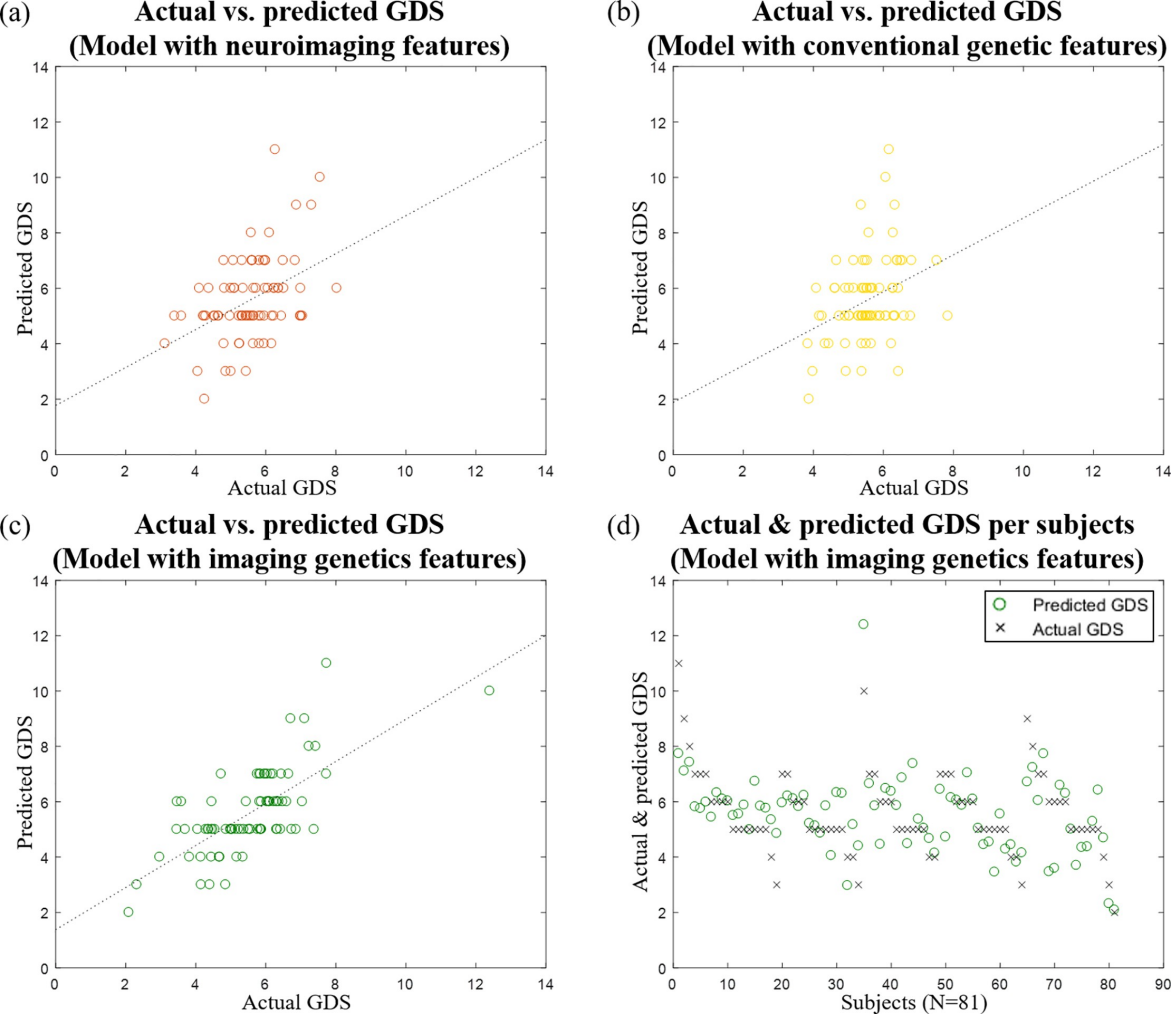
The prediction plots of the three models. (a), (b), and (c) show the actual and predicted GDS from Models using neuroimaging features, conventional genetic features, and imaging genetics features, respectively. The dashed line indicates the identity line. (d) shows the actual GDS and predicted GDS for each subject using our proposed model using imaging genetics features (N = 81).

The three linear regression models were constructed using features from imaging genetics, structural connectivity, and conventional references to predict GDS in a five-fold cross-validation. The first model of imaging genetics was our proposed model and it jointly considered imaging and genetic features. Our proposed model using imaging genetics features showed meaningful correlation (r = 0.749, p = 0.001; averaged) between the predicted and real GDS over five left out test folds. The mean RMS error between the predicted and actual GDS was 0.991 (standard deviation [SD] 0.242). The second model used only imaging features and showed a moderate correlation and RMS error (r = 0.371, p = 0.175, RMS error = 1.370 [SD 0.329]; averaged) between the predicted and real GDS. The model using only the reference-based genetic features achieved the correlation RMS error that were similar to the model with only neuroimaging features (r = 0.278, p = 0.157, RMS error = 1.452 [SD 0.296]; averaged). The prediction plots of the three models are given in Fig 2. We found that the adjusted R^2^ values for models using only imaging features and the reference-based genetic features were less than 0.3, while the adjusted R^2^ for our proposed model was larger than 0.6 over five training folds. We confirmed that genetic features derived from the intermediate phenotype of imaging features could provide complementary information to explain the degree of depression (determined by GDS), whereas imaging features and genetic features used alone contributed less. The features adopted in these three models came from different sources of information. Combining the models could provide complementary information, which may lead to an improved explanation of GDS. There are three sets of features and there could be many distinct combinations of the feature sets. Detailed results from the all possible combined models are shown in Figures A and B in S1 File.

We identified SNPs that could explain GDS well using the imaging genetics approach limited to PD patients. It is still possible that the identified SNPs could also be present in NC participants using the same approach. We applied the same imaging genetics approach to NC participants (n = 69) from the PPMI database. Unfortunately, no imaging features that explained GDS well were computed from the NC group. This led to applying the imaging genetics approach infeasible as there were no intermediate phenotypes available. If the SNPs identified in the PD group were indeed present in the NC group, the SNPs should be able to explain GDS well in the NC group as well. We applied the linear model of the identified SNPs learned from PD to the NC group. The results showed that the identified SNPs were not effective at explaining the degree of depression in the NC group with a very low adjusted R2 value (i.e., < 0.01, closet to zero). Taken together, we believe the identified SNP were only effective at explain GDS within PD group and thus the features could be specific to DPD.

## Discussion

Psychological phenomena are difficult to characterize using neuroimaging or genetic analysis alone as the given diagnosis spans a wide spectrum of symptoms. Some argue that diagnosis is becoming less relevant due to this problem [41,42]. The DPD diagnostic criteria used in this study were also affected by this problem. Depression occurs in approximately 40% of PD patients and DPD and nDPD patients share many symptoms such as cognitive decline, motor impairment, and helplessness, which makes separating DPD from nDPD difficult [6,7,43]. Depression is multifactorial and its manifestation varies significantly when it accompanies neurodegenerative diseases [5–8,44]. Therefore, in this study, we constructed models to predict the degree of depression in PD patients rather than the diagnosis of depression. We identified several features that indicate the degree of depression in PD using the imaging genetics approach. The features were used to predict the degree of depression and the performance was enhanced when features derived from imaging genetics were used. If each of the resulting SNPs from imaging genetics is studied in more detail, these SNPs could be used as biomarkers related to DPD in the future.

Imaging genetics analysis is better than conventional analysis because imaging observations are used as intermediate phenotypes [16]. Others have adopted psychiatric or behavior intermediate phenotypes and have reported improved biological characterization and validation of genetic effects [45]. Imaging genetics can describe a neural system that is affected by genetic variation and identify polymorphisms beyond a simple statistical association. Imaging genetics is a powerful bottom-up approach to elucidate biologically valid knowledge of previously unknown mechanisms such as the detailed mechanism of psychiatry. Therefore, imaging genetics could be used for the discovery of neural circuits that convert genetic influences into behavior. These imaging genetics approaches might enable us to further understand the neurobiology of DPD.

We identified 18 SNPs related to eight regional structural connectivity measures that contributed to predicting the depression-related score (i.e., GDS) in PD patients. The SNPs were matched with genes. Among the identified genes, several genes were overexpressed in the identified ROIs as intermediate phenotypes. NRXN1 and LOC284395 are known to be related to the anterior cingulate cortex [37]. LOC284395 is also related to amygdala [37]. The ARHGAP24 and RAB15 are known to affect hippocampus [46,47]. The structural connectivity in the anterior cingulate cortex, hippocampus, and amygdala showed significant contributions to predicting the GDS score. The identified regions are involved in the corticolimbic system [48,49], and previous studies revealed limbic structures were related with DPD [50,51]. The corticolimbic system is known as an important pathway associated with the dopamine secretion [48,52,53]. Similar to our results, previous studies already revealed limbic structures are closely related with DPD patients, regardless of neurotransmitter. Even though the exact mechanism of DPD patients was fully understood, dopaminergic medications improve DPD patients and dopaminergic involvements could be considered based on our results [54]. In addition to the dopaminergic neurotransmission system, the neurodegenerative processes in PD patients involve the serotonergic or cholinergic system [53], and the limbic structures including hippocampus and amygdala were associated with serotonin and acetylcholine [15,55,56] that affects dopamine deficit and increases the risk of depression [57]. Taken together, the identified genes might affect the structural connectivity of various structures such as anterior cingulate cortex, amygdala, and hippocampus, which might alter the degree of depression in PD via many neurotransmitter systems. The possible downstream pathway from involved SNP/gene is left for future work as there is a very limited literature associating SNP/gene with depression related pathways. Interestingly, our imaging genetics model identified 18 SNPs and they did not overlap with the three SNPs identified in the reference-based model. We were able to identify the three SNPs if we relaxed the first selection procedure with an empirical p-value threshold of 0.05 and the constraint of association with two or more imaging features.

In addition to the regions in the corticolimbic system, we identified postcentral gyrus, precuneus, and temporal pole. These regions were also reported to be related to DPD patients [7,12,58]. The alteration in postcentral gyrus was associated with the cortical-basal ganglia circuit that is known as an important system that controls motor symptoms in PD patients [59]. The precuneus is generally known as the region highly connected with the posteromedial cortex [33]. It is known to be involved in motivation, planning, and social behavior and part of the default mode network which can be altered in depressive patients [60]. *Krug et al*. found that the left precuneus was more activated in healthy subjects carrying a specific gene found to be overrepresented in patients suffering from bipolar disorder, depression, or schizophrenia [60,61]. The temporal pole is heavily involved in facial emotion processing [62] that is known to be strongly linked with depression [63]. Based on our results, the SNP/Gene in our study may be associated with various neuronal circuits with many neurotransmitter systems. Therefore, with disease progression, PD patients may present with diverse motor and non-motor symptoms, including depression, from involvements of aforementioned systems.

We used DC of structural connectivity because psychiatric disorders such as depression were thought to be related to the complex interactions of the brain regions, and thus we considered a more network-oriented complex measure such as DC as the intermediate phenotype compared to simple measures such as fractional anisotropy (FA) and mean diffusivity (MD). In the current study, we found larger DC values in DPD patients compared to the nDPD patients (Table 2). Among the identified regions, hippocampus, amygdala, postcentral gyrus, and temporal pole showed significant between-group differences in DC values between DPD and nDPD groups. The hippocampus and amygdala are involved in the limbic system that regulates emotion and memory; thus, abnormalities in these regions appear to contribute to the pathophysiology of mood disorders [64]. A previous study observed that the depressed patients showed microstructural alteration in the hippocampus in terms of FA and MD [65] indicating the structural connectivity of hippocampus could be altered in depressed patients. One study explored the structural connectivity in the PD patients with sleeping behavior disorder that is highly correlated with depression [66,67]. They reported increased centrality in the patients of sleeping behavior disorder in PD in the amygdala and hippocampus compared to the patients without sleeping behavior disorder in PD [66]. Those studies were collectively consistent with our results in that the regions in the limbic system are related to depression. We could not find prior studies related to postcentral gyrus and temporal pole. Further studies are needed in these regions to properly study the DPD.

We conducted additional experiments to see if the imaging features were related to clinical scores provided by the PPMI database. If the imaging features were affected by other psychiatric disorders, they could be used to the predict clinical score related to the psychiatric disorder. We predicted STAI, MoCA and QUIP scores using the same imaging features in a regression framework. The models that predicted MoCA and QUIP scores showed very low correlations (r = -0.040, p = 0.221; r = 0.074, p = 0.164; averaged; QUIP and MoCA, respectively) between the predicted and actual scores. When STAI was predicted with the same imaging features, we observed a slightly lower correlation (r = 0.225, p = 0.188; averaged) than the existing correlation with GDS (r = 0.371, p = 0.175; averaged). This is consistent with literature that depression and anxiety are linked strongly, and they co-occur frequently [68]. This confounding issue needs to be validated with future studies with carefully curated data.

Imaging genetics is a sensitive analysis for identifying additional genetic information that impacts brain function [16]. Still, the SNPs we identified in this study need further validation using independent large-scale cohorts because most genes expressed in the brain are likely to have variable effects and the link between the identified SNPs and depression could be weak. The results of our study need to be interpreted in the context of its limitations. Our study is also limited by the small number of samples. This is mainly because we used data from a research database. This issue was partly mitigated by two-tiered selection approaches, where only a small number of SNPs were selected for the models. Our findings need to be validated on a larger cohort in the future. The imaging data in our study were obtained from nine different sites, but all nine sites adopted the same image acquisition protocol using the same MRI scanner model (Siemens 3T scanner) to reduce confounding effects. In addition, we added multi-center information as a nuisance covariate to account for possible confounding effects in the computation of DC derived from structural connectivity analysis. Despite many previous studies, various neurotransmitters were reported to be involved in PD patients and the mechanism of depression in PD patients has not been fully understood yet. Depression in PD and non-PD patients could be clinically different. Depression is a very heterogeneous disorder and also common in non-PD patients. Depression is already known as a pre-motor symptom for PD [69] and related with prognosis in PD patients [70]. Furthermore, unlike the depression in non-PD subjects, the dopaminergic system is regarded to be involved and dopaminergic medication could be helpful for the DPD patient [54].

We combined two types of distinct information to predict the degree of depression. The genetic information is rather fixed, but imaging could be performed depending on the patient’s condition. Our model combining imaging and genetics information could be applied whenever a patient undergoes new imaging and thus could be used for the early prediction of depression. If detected, patients could be directed to many non-drug therapy options that are only available in the early stages of depression. Our study is also the first to propose a model that can explain the degree of depression (determined by GDS) in PD using diffusion MRI and SNP data.

## Supporting information

S1 File The validation and prediction results of combining the three models in main text. This S1 File included the validation and prediction results of constructed additional models by integrating different combinations of our proposed model using imaging genetic features, the model using only neuroimaging features, and the model using only conventional genetic features.
(DOCX)
