# Supplementary material for: Effectiveness of imaging genetics analysis to explain degree of depression in Parkinson’s disease
Source: PLoS One. 2019 Feb 11;14(2):e0211699. doi: 10.1371/journal.pone.0211699 (PMC6370199; doi:10.1371/journal.pone.0211699)
Supplement: S1 File — This S1 File included the validation and prediction results of constructed additional models by integrating different combinations of our proposed model using imaging genetic features, the model using only neuroimaging features, and the model using only conventional genetic features. (DOCX) [file pone.0211699.s001.docx]

**Supplementary Information**

**Title: Effectiveness of imaging genetics analysis to explain degree of depression in Parkinson’s disease**

Authors: Ji Hye Won^1,2^, Mansu Kim^1,2^, Bo-yong Park^1,2^, Jinyoung Youn^3,4^ and Hyunjin Park^2,5^*

^1^ Department of Electrical and Computer Engineering, Sungkyunkwan University, Suwon, Korea
^2^ Center for Neuroscience Imaging Research, Institute for Basic Science, Suwon, Korea

^3^ Department of Neurology, Sungkyunkwan University School of Medicine, Samsung Medical Center, Seoul, Korea

^4^ Neuroscience Center, Samsung Medical Center, Seoul, Korea

^5^ School of Electronic and Electrical Engineering, Sungkyunkwan University, Suwon, Korea

*Corresponding Author

Hyunjin Park Ph.D.
Center for Neuroscience Imaging Research

School of Electronic and Electrical Engineering
Sungkyunkwan University, Suwon, Korea, ZIP: 16419
Tel: +82-31-299-4956

Fax: +82-31-290-5819

Email: hyunjinp@skku.edu

**Supplementary results**

**Validation and prediction performance of combining different models**

We constructed additional models by integrating different combinations of our proposed model using imaging genetic features, the model using only neuroimaging features, and the model using only conventional genetic features. We assigned numerical designation for the three models for notational convenience. The model using only neuroimaging features was denoted as model 1, the model using only conventional genetic features was denoted as model 2, and our proposed model using imaging genetic features was denoted as model 3. We compared total of seven models, including the three models mentioned in the main text: the model combining models 1 and 2 (denoted as the fourth model); the model combining models 2 and 3 (as the fifth model); the model combining models 1 and 3 (as the sixth model); and the model combined models 1, 2, and 3 (as the seventh model). We confirmed the tendency of the adjusted R^2^ value to increase as we combined different models containing more features over five training folds. The models achieved over 0.6 adjusted R^2^ value only if we included model 3 (i.e., the model using imaging genetic features). Compared to model 3 using only imaging genetics analysis we proposed, adding genetic and imaging information to the model 3, the adjusted R^2^ did not increase significantly.

We evaluated the performance of all seven models (three main models and additional combined models) over five test folds. RMS error and the results of correlation (the value of r and p) between the predicted and actual GDS scores for the seven models are shown in Supplementary Figure A. The prediction plots of all models are given in Supplementary Figure B. In the previous section, we observed better fitting in terms of adjusted R^2^ as we included more features. For validation and prediction, the third model using only imaging genetics showed the lowest RMS error (0.9910; averaged) and best correlation (r = 0.7486, p =0.0012; averaged). This might imply that imaging genetics analysis is the most suitable for creating a prediction model.


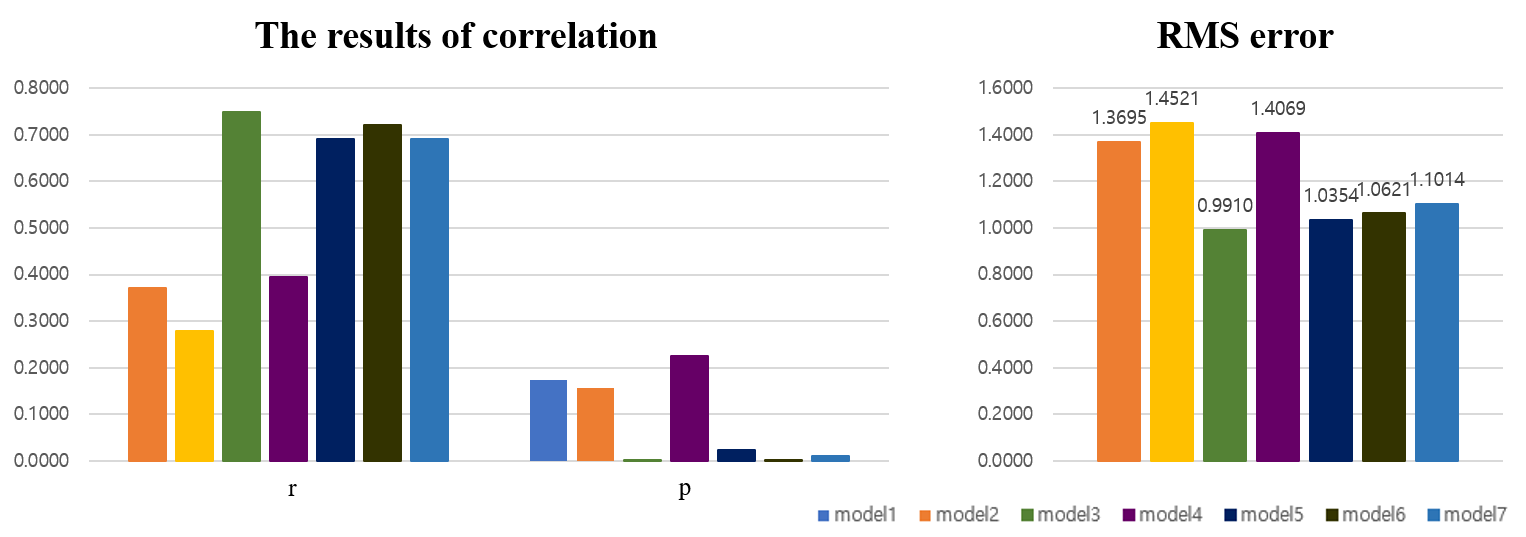


Supplementary Figure A. The comparison of seven models with correlation results and RMS error between the predicted and actual GDS scores.


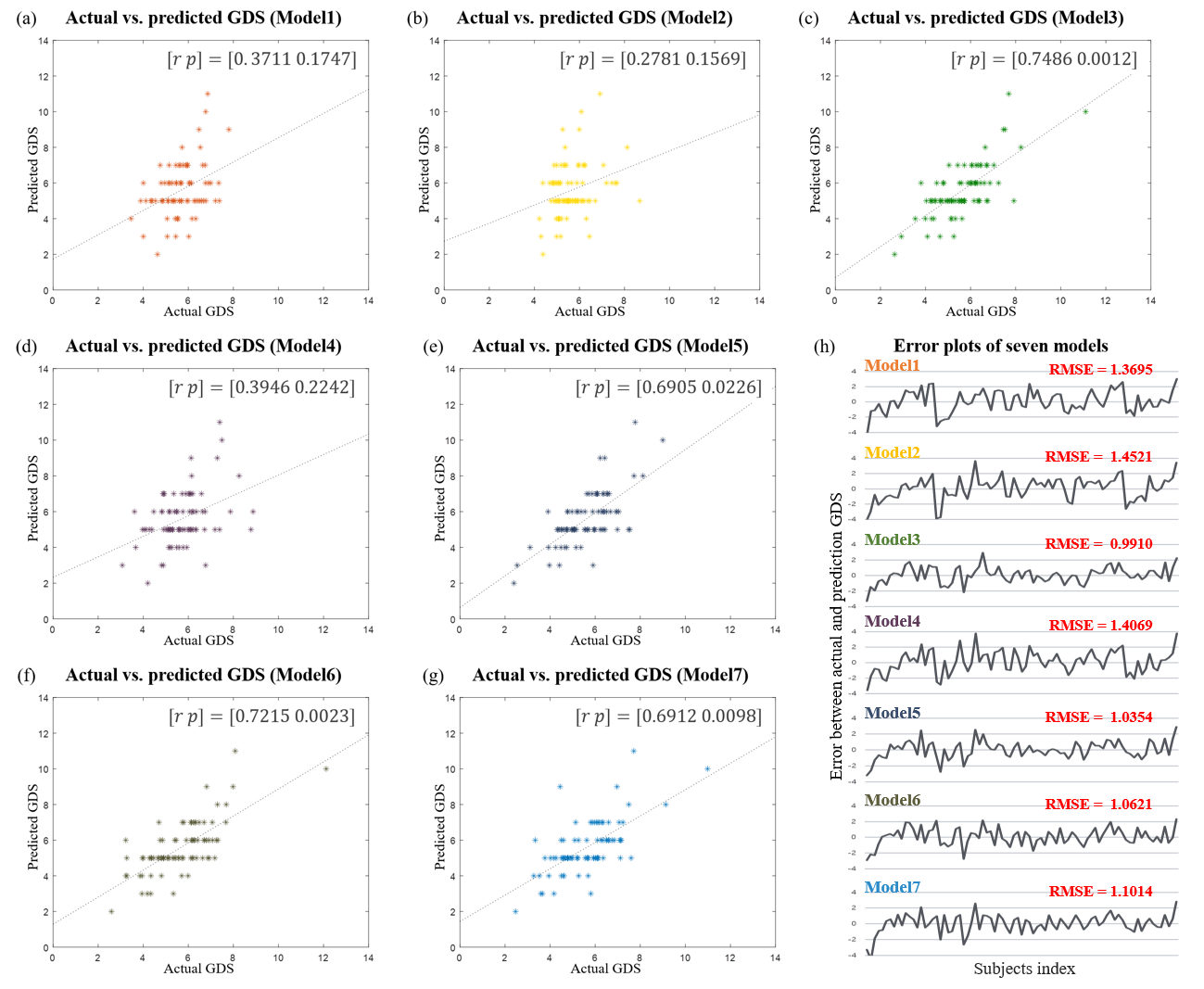


Supplementary Figure B. The prediction plots of seven models. (a)-(g) show the actual and predicted GDS from Models 1-7, respectively. The dashed line indicates the identity line. (h) shows error plots of the seven models.
